# A Case of Warm Autoimmune Hemolytic Anemia Secondary to Epstein-Barr Virus Infection

**DOI:** 10.7759/cureus.26371

**Published:** 2022-06-27

**Authors:** Oluseyi Abidoye, Comfort Adewunmi, Shravanti Macherla

**Affiliations:** 1 Internal Medicine, Northeast Georgia Medical Center Gainsville, Gainesville, USA; 2 Hematology and Oncology, Longstreet Cancer Center, Gainesville, USA

**Keywords:** immune hematology, epstein-barr virus, viral infection, warm autoimmune hemolytic anemia, autoimmune hemolytic anemia (aiha)

## Abstract

Autoimmune hemolytic anemia (AIHA) is a rare disease characterized by autoantibodies directed at red blood cells. Patients typically present with anemia and are diagnosed by positive direct antiglobulin (DAT) test. AIHA is subclassified into warm or cold based on antibodies involved and depending on their optimal temperature in which they react with RBC antigens. Warm AIHA can be either primary (idiopathic) or secondary depending on etiology. Secondary causes are associated with malignancy, connective tissue and inflammatory diseases, infections (typically viral infections), or drugs (e.g., antibiotics, chemotherapeutic agents). Epstein-Barr virus (EBV) is a herpes virus that is commonly associated with cold AIHA, with only one reported case of EBV-induced warm AIHA. It has been postulated that antibodies against EBV cross-react with antigens expressed on RBC membranes and activate the complement cascade similarly. This case report describes a case of a 32-year-old male who presented with warm AIHA secondary to EBV reinfection.

## Introduction

Autoimmune hemolytic anemia (AIHA) is a rare disease characterized by autoantibodies directed at red blood cells (RBCs) [1]. AIHA is subclassified into warm or cold based on antibodies involved and depending on their optimal temperature in which they react with RBC antigens [1]. Warm AIHA can be either primary (idiopathic) or secondary depending on etiology [1]. Secondary causes are associated with malignancy, connective tissue and inflammatory diseases, infections (typically viral infections), or drugs (e.g., antibiotics, chemotherapeutic agents). [1] Epstein-Barr virus (EBV) is a herpes virus that is commonly associated with cold AIHA, with only one reported case of EBV-induced warm AIHA. [2] This case report describes a case of a 32-year-old male who presented with warm AIHA secondary to EBV reinfection.

## Case presentation

In November 2021, a 32-year-old Hispanic male with a previous medical history of COVID infection in October 2021 presented to our institution with generalized weakness, occasional shortness of breath on exertion, intermittent fevers, and intermittent left knee pain and swelling for one-month duration. He was seen by his primary care provider for the left knee pain and swelling prior to presentation. He underwent arthrocentesis prior to presentation, which was remarkable for uric acid crystals with cytology significant for neutrophilic predominance with negative cultures. He received two rounds of prednisone; however, his left knee pain and swelling persisted with his pain unbearable as the patient was unable to bear weight comfortably.

He denied any prior history or family history of hemolytic anemia. He also denied any new medications except for steroids and ibuprofen. He denied any history of smoking or alcohol use or illicit drug use.

**Figure 1:**
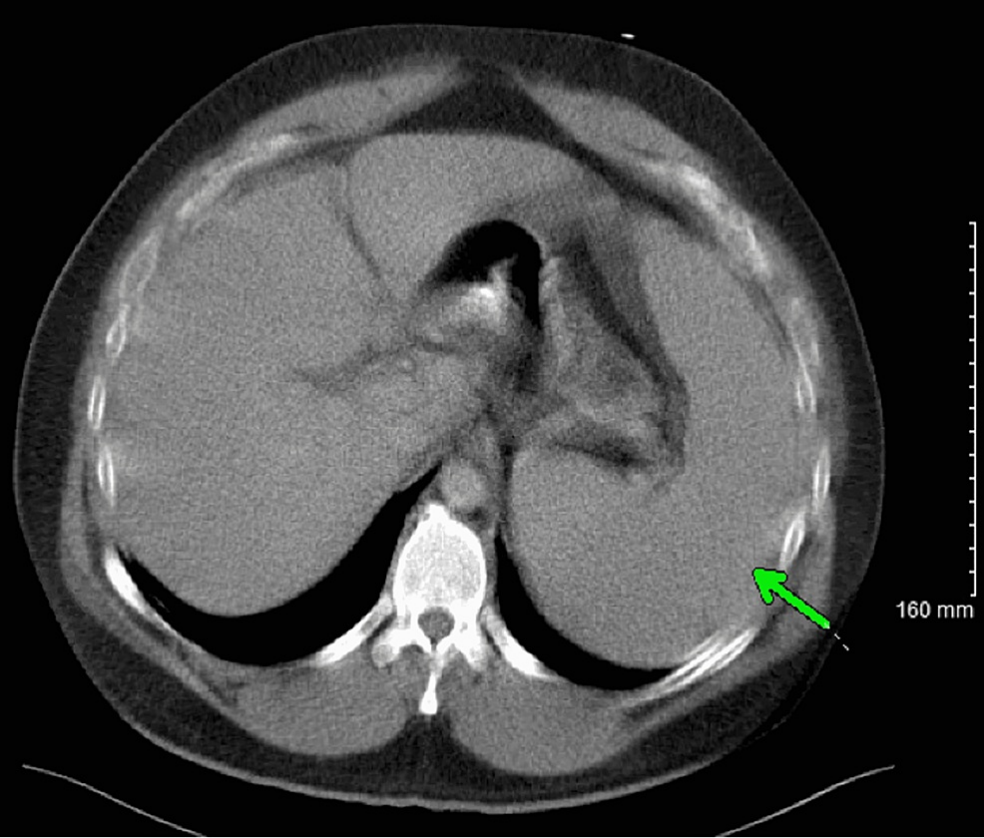
Computed tomography (CT) of the abdomen, with axial view showing (green arrow) splenomegaly

On admission, he appeared fatigued and pale. He was tachycardic and tachypneic but afebrile. There was no palpable lymphadenopathy. In addition, he also noted to have left knee swelling, which was tender on palpation, warm to touch, and erythematous with effusion on examination. All investigations conducted during hospitalization are given in Table 1. His complete blood count (CBC) showed a white blood cell count of 3.3 K/uL, hemoglobin of 7.5 g/dL, and platelet count of 179 K/uL. Further evaluation showed absolute neutrophil count of 0.66 x 10^3^/uL, reticulocyte count of 9.6 % (absolute reticulocyte count: 0.25 x 10^6^/uL), lactate dehydrogenase (LDH) of 268 U/L, haptoglobin of 2 mg/dL, total bilirubin of 2.20 mg/dL, and direct bilirubin of 0.6 mg/dL. The direct antiglobulin test was positive for IgG and C3. Antibody screenings PEG (polyethylene glycol) and LISS (low ionic strength saline) were positive, and eluate was reactive. Antibody ID test revealed the presence of warm autoantibody. Cold agglutinin titer levels were 1:32. Peripheral blood smear revealed normocytic anemia and reactive lymphocytosis. Anti-nucleic acid titer was 1:40, rheumatoid factor was negative. HIV and hepatitis tests were negative. serum EBV antibodies to nuclear antigen, early (D) antigen, and capsid (IgG and IgM) were elevated. Bone marrow biopsy was performed, which was negative. Computed tomography of the chest, abdomen, and pelvis revealed massive splenomegaly and small mesenteric and retroperitoneal lymph nodes measuring less than 1 cm (Figure 1). Based on the results, the patient was diagnosed with warm AIHA. He was started on a course of steroids (prednisone 1 mg/kg and rituximab), with consequent improvement in his hemoglobin. Table 2 shows serial hemoglobin, LDH, haptoglobin, reticulocyte count, and rituximab doses. Regarding his left knee swelling, swelling improved with steroids. He was discharged to follow-up outpatient with his hematologist and rheumatologist. He received four doses of rituximab weekly and remained on steroid maintenance. He had a six-month follow-up, which showed improvement with laboratory response.

**Table 1:** Summary of baseline investigations with laboratory reference ranges MCV, mean cell volume; LDH, lactate dehydrogenase; Ig, immunoglobulin; ESR, erythrocyte sedimentation rate; RNP, ribonucleoprotein; SS, Sjogren's antibodies; Sm, Smith antibodies; EBV, Epstein-Barr Virus; PCR, polymerase chain reaction; DTA, direct antiglobulin test

Investigation	Result and units	Reference range
Hemoglobin	7.5 g/dL	14-18
White blood cells	3.3 K/uL	4.8-10.8
Platelets	179 K/uL	130-400
Absolute neutrophil count	0.66 10^3^/uL	2-8.1
Absolute lymphocyte count	1.35 10^3^/uL	0.75-5.5
Absolute monocyte count	0.99 10^3^/uL	0-1.2
Atypical lymphocytes	1 %	
Bands	13 %	0-3
MCV	89.4. fL	80-94
Reticulocytes	0.25 10^6^/uL	0.03-0.10
Haptoglobin	2.00 mg/dL	30-200
LDH	268 U/L	84-246
B12	636 pg/mL	193-986
Folate	7.82 ng/mL	>2.8
Sodium	135 mmol/L	135-148
Potassium	4.1 mmol/L	3.5-5.2
CO_2_	26 mmol/L	21-32
Chloride	102 mmol/L	100-110
Urea	22 mg/dL	3-23
Aspartate aminotransferase	27 U/L	0-48
Alanine aminotransferase	56 U/L	13-61
Total protein	8.2	6.0-8.3
Albumin	4.4 g/dL	3.4-5
Total bilirubin	2.20 mg/dL	0-1.00
Direct bilirubin	0.6 mg/dL	0-0.4
Creatinine	0.97 mg/dL	0.8-1.30
Calcium	9.2 mg/dL	8.4-10.6
Alkaline phosphatase	118 U/L	45-136 U/L
Unconjugated bilirubin	1.6 mg/dL	<0.6
ESR	34 mm	0-15 mm
C-reactive protein	6.90 mg/dL	0-0.60
D-dimer	1.33 ug/mL FEU	≤0.400
Anti-DNA antibody, double-stranded	<12.3 (negative) IU/mL	<30.0
RNP antibodies, IgG	0.3	< 1.0
SS-A antibodies, IgG	<0.2	<1.0 U
SS-B antibodies, IgG	<0.2	<1.0 U
Chromatin (nucleosomal) antibody	< 1.0 NEG	< 1.0 NEG
Sm Ab, IgG	<0.2	< 1.0 U
Centromere antibodies, IgG	<0.2	< 1.0 U
Mononucleosis screen	Negative	
Cold agglutinin screen	<1:32	<1:32
Hepatitis panel	Negative	
Hepatitis B surface antibody	Positive	
HIV1 + 2 AB + P24G	Nonreactive	
EBV by PCR	Not detected	
Peripheral smear		
Direct Coombs test	DAT positive ( DAT IgG Gel and C3)	
Bone marrow aspirate immunophenotyping	Normocellular trilineage hematopoiesis with maturation was negative for leukemia, lymphoma, plasma cell dyscrasia, and extrinsic tumor. Erythroid hyperplasia with myeloid: erythroid ratio of 0.9:1	
CT of the thorax/abdomen/pelvis	Splenomegaly measuring 21.7 cm in longest diameter. Small inguinal, mesenteric, and retroperitoneal lymph nodes measuring less than 1 cm in diameter	
EBV antibody to nuclear antigen IgG	>600.0 high	0.0-21.9 U/mL
EBV antibody to early (D) antigen IgG	22.8	0.0-10.9 U/mL
EBV capsid IgG antibody	>750.0 positive	0.0-21.9 U/mL
EBV capsid IgM antibody	76.6 high	0.0-43.9 U/mL

**Table 2:** Summary of laboratory response during the treatment course with laboratory reference ranges

Treatment duration	Hemoglobin (g/dL)	Haptoglobin (mg/dL)	Lactate dehydrogenase (U/L)	Reticulocyte count (10^6^/uL)
1 week after treatment	10	2	268	0.25
2 weeks later	9.7	58	191	0.3
4 weeks later	12.0	80	150	0.1

## Discussion

AIHA is a rare form of hemolysis resulting from the host’s system attack on its red cell antigens [1]. It is an immune disease characterized by RBC destruction due to autoantibodies acting against RBC antigens with or without complement activation [3]. Hemolytic anemia results if RBC destruction or hemolysis occurs at a rate at which the body cannot compensate. Hemolysis can either be acute or chronic depending on onset. Patients with AIHA typically have common laboratory findings such reticulocytosis, elevated unconjugated bilirubin and LDH, serum aspartate aminotransferase disproportionately higher than serum alanine aminotransferase, and decreased haptoglobin [1].

The antibodies involved in AIHA are detected on the RBC surface using the direct Coombs test or DAT test [1]. AIHA can be classified based on optimal temperature in which the autoantibodies are able to bind to RBC antigens in vivo - warm AIHA or cold AIHA - further subdivided into cold agglutinin disease and paroxysmal cold hemoglobinuria (PCH) [3]. There have been several reported cases of mixed-type picture involving warm and cold AIHA [3]. Cold AIHA and PCH involve cold-reacting autoantibodies that are maximally reactive in cold temperatures ranging from 1 to 3 degrees Celsius. Cold AIHA involves IgM antibodies directed against I/i RBC antigens, while PCH involves IgG antibodies directed against P antigens of RBCs [3].

Warm AIHA involves IgG antibodies with maximal reactivity at body temperature. It can either be primary - not associated with underlying cause or secondary due to underlying cause. About half of warm AIHA cases are primary in nature, while secondary warm AIHA accounts for the remaining 50% of cases [4]. Secondary warm AIHA has been associated with chronic conditions such connective tissue disorders, immunodeficiency syndrome, or hematological malignancies such as chronic lymphoblastic leukemia (CLL), non-Hodgkin’s lymphoma, and solid tumors; medications; antibiotics and chemotherapeutic agents; infections; mostly virals such as cytomegalovirus, HIV, hepatitis C, varicella, mumps, rubella, and influenza; or previous transplantation or transfusion [3,4]. Our patient demonstrated warm autoantibodies likely due to EBV infection. The serological investigations of a positive anti-EBV capsid IgG antibody and anti-EBV capsid IgM antibody in our patient likely suggested reinfection [5]. We were able to exclude other causes of AIHA by undergoing a thorough rheumatology and infectious workup, which were all unremarkable. Given the results, we deduced that the likely etiology for warm AIHA was due to EBV infection, which is rare.

Cold AIHA has been extensively linked with EBV infection and as stated earlier involves IgM antibodies that attack I-antigens on RBCs [1]. We were able to detect warm IgG antibodies in our patient with EBV infection rather than cold IgM antibodies typically associated with EBV infection. It has been reported that primary EBV infections are associated with AIHA in about 3% of cases, with most cases being mild in nature [1]. Our patient had positive warm antibodies, which is unique. An extensive literature review showed only one single report of fatal AIHA due to IgG warm agglutination induced by EBV infection [6]. We believe that AIHA in our patient is likely due to reactivation of latent EBV infection. The mechanism by which AIHA develops from EBV infection is unclear. Several theories have been postulated, with one possibility that antibodies against EBV cross-react with antigens expressed on RBC membranes and activate the complement cascade similarly [7].

Regarding treatment, first-line treatment involves steroids (1 mg/kg of prednisone orally or intravenous methylprednisolone) and transfusion depending on the severity of anemia. If there is no steroid response, rituximab can be considered a second-line agent [7,8]. Our patient received both steroid and rituximab with significant response. Patients with AIHA have been reported to have good prognosis with timely management [7]. Regardless of good prognosis, patients have a high probability of relapse even after steroid therapy [7,8].

## Conclusions

We reported a case of EBV-exacerbated warm AIHA. Cold agglutin AIHA has been reported to be associated with EBV infection, with only one reported case of EBV-induced warm AIHA. This case highlights the fact that a reactivation of a latent EBV infection may exacerbate a warm AIHA, which can lead to life-threatening acute hemolysis if prompt treatment is not provided. Further studies are needed to delineate causation.
